# Clinical Phenotypes Associated with the Gut Microbiome in Older Japanese People with Care Needs in a Nursing Home

**DOI:** 10.3390/nu16223839

**Published:** 2024-11-08

**Authors:** Rikako Inoue, Koji Hosomi, Jonguk Park, Haruka Sakaue, Hitomi Yumioka, Hiroko Kamitani, Yoshiharu Kinugasa, Kaori Harano, A. Yasmin Syauki, Miki Doi, Suzumi Kageyama, Kazuhiro Yamamoto, Kenji Mizuguchi, Jun Kunisawa, Yasuyuki Irie

**Affiliations:** 1 Department of Nutritional Science, Faculty of Health and Welfare Science, Okayama Prefectural University, Soja City 719-1197, Okayama, Japan; rinoue@fhw.oka-pu.ac.jp; 2 Microbial Research Center for Health and Medicine, National Institutes of Biomedical Innovation, Health and Nutrition (NIBIOHN), Ibaraki City 567-0085, Osaka, Japan; hosomi@omu.ac.jp (K.H.); yumioka@osaka-seikei.ac.jp (H.Y.); kunisawa@nibiohn.go.jp (J.K.); 3 Graduate School of Veterinary Science, Osaka Metropolitan University, Izumi-Sano City 598-0048, Osaka, Japan; 4 Artificial Intelligence Center for Health and Biomedical Research, National Institutes of Biomedical Innovation, Health and Nutrition (NIBIOHN), Ibaraki City 567-0085, Osaka, Japan; jonguk@nibiohn.go.jp (J.P.); kenji@protein.osaka-u.ac.jp (K.M.); 5 Graduate School of Health and Welfare Science, Okayama Prefectural University, Soja City 719-1197, Okayama, Japan; skaaue71@gmail.com (H.S.); syaukiyasmin@gmail.com (A.Y.S.); doimiki1214@gmail.com (M.D.); suzumi.kageyama@gmail.com (S.K.); 6 Faculty of Nutrition, Osaka Seikei College, Osaka City 533-0007, Osaka, Japan; 7 Department of Cardiovascular Medicine and Endocrinology and Metabolism, Faculty of Medicine, Tottori University, Yonago City 683-8503, Tottori, Japan; hirokoro126@yahoo.co.jp (H.K.); ykinugasa-circ@tottori-u.ac.jp (Y.K.); ykazuhiro@tottori-u.ac.jp (K.Y.); 8 Department of Human Welfare, Faculty of Human Relations, Otsuma Women’s University, Tama City 206-8540, Tokyo, Japan; harano@otsuma.ac.jp; 9 Department of Nutrition, Faculty of Medicine, Hasanuddin University, Tamalanrea 90245, Makassar, Indonesia; 10 Research Fellow of Japan Society for the Promotion of Science, Chiyoda City 102-0083, Tokyo, Japan; 11 Institute for Protein Research, Osaka University, Suita City 565-0871, Osaka, Japan

**Keywords:** frailty, microbiome, poor nutrition, clinical phenotype, nursing home

## Abstract

Background: Frailty increases the risk of needing nursing care and significantly affects the life and functional prognosis of older individuals. Early detection and tailored interventions are crucial for maintaining and enhancing their life functions. Recognizing distinct clinical phenotypes is essential for devising appropriate interventions. This study aimed to explore diverse frailty phenotypes, focusing on poor nutrition in older Japanese individuals through observational research. Methods: Twenty-one nursing home residents underwent a comprehensive survey covering physical, blood, dietary, cardiac, cognitive, nutritional, nursing care, frailty, agitated behavior, and gut microbiome assessments (high-throughput 16S rRNA gene sequencing). Using clustering analysis with 239 survey items (excluding gut microbiome), participants were classified into subgroups based on clinical phenotypes, and group characteristics were compared through analysis. Results: Individuals with moderate or severe frailty and suspected dementia formed subgroups with distinct clinical phenotypes based on nutritional, defecation, and nursing care statuses. The gut microbiome significantly varied among these groups (*p* = 0.007), indicating its correlation with changes in clinical phenotype. Nutritional status differences suggested poor nutrition as a differentiating factor in the core clinical phenotype. Conclusions: This study proposes that the gut microbiome differs based on the clinical phenotype of Japanese older individuals with frailty, and targeted interventions addressing the gut microbiome may contribute to preventing frailty in this population.

## 1. Introduction

According to the United Nations’ World Population Prospects, the global population aged 75 and older is experiencing significant growth. As of 2024, approximately 9.2% of the world’s population is aged 65 and above, with a notable increase projected for those aged 75 and over. Specifically, the population aged 80 and above is expected to more than triple between 2024 and 2074, illustrating a dramatic shift in demographic structure. This aging trend is influenced by longer life expectancies and declining fertility rates, which are prevalent across both developed and developing nations. In Japan, the aging of the population is advancing at an unprecedented rate. Projections indicate that the population of elderly individuals (those aged 75 and older) was 15.5% of the total population in 2022 and is expected to rise to 25.1% by 2070 [1]. Notably, the decline in the autonomy of the oldest-old (aged 85 and older) is particularly striking, with estimates suggesting that this group will surpass 10 million nationwide by 2035. The increasing proportion of individuals in this age bracket entering nursing homes highlights the growing importance of health management for the elderly [2].

Frailty, which significantly impacts the healthy lifespan of older adults, is broadly defined as a state of increased vulnerability to external stressors, resulting from the cumulative decline in multiple physiological systems due to aging [3,4]. As sarcopenia (muscle weakness) progresses, the risk of falls, hospitalization, and dependency increases, making the prevention of frailty a pressing public health concern [5]. Frailty arises from various factors, including malnutrition, reduced physical activity, and chronic diseases, highlighting the importance of comprehensive and sustainable management for its prevention. In Japan, initiatives to strengthen the management system are being supported by concurrent revisions to medical and long-term care fees [6].

Recent studies have increasingly highlighted the relationship between the gut microbiome and frailty. The gut microbiome is crucial in regulating digestive function, immune response, and metabolic processes, and there is growing evidence that dysbiosis of the gut microbiome may contribute to malnutrition and frailty [7]. For instance, the research conducted in East Africa has demonstrated a link between gut bacteria and malnutrition-related conditions such as kwashiorkor [8], suggesting that the gut microbiome may also play a role in the onset of malnutrition among other populations.

Furthermore, particular attention must be given to elderly individuals residing in nursing homes. This demographic is more likely to experience reduced physical activity, dietary restrictions, chronic diseases, and medication use, which may lead to more profound alterations in the composition and functionality of the gut microbiome compared to those living independently. The extent to which these changes in the gut microbiome contribute to the progression of frailty remains an area requiring further investigation [9].

The objective of this study is to elucidate the association between the gut microbiome and frailty in elderly residents of nursing homes and to explore potential intervention strategies aimed at preventing and mitigating frailty.

## 2. Materials and Methods

### 2.1. Participants

The study population included 24 older individuals (age, 68–101 [86.5 ± 8.5] years) who were admitted to a single nursing home (34°38′28.2366″, 133°41′8.5452″). The researcher explained the purpose of this research and obtained consent of the participant or their substitute. Since three individuals withdrew during the study, a total of 21 older individuals (male, *n* = 5; female, *n* = 16) were ultimately analyzed. Data collection was conducted from January 2017 to September 2022. The participant characteristics are shown in Table 1. We chose to focus on nursing home residents because their clinical characteristics and lifestyle habits, including dietary patterns, differ significantly from those of healthy individuals, which is closely related to variations in the gut microbiome. The nutritional risk, according to the GNRI, was classified as follows: no risk (*n* = 2), mild (*n* = 3), moderate (*n* = 10), and severe (*n* = 6). We confirmed that the subjects had not taken antibiotics from one month before the start of the study until the end of sampling.

The researchers explained the purpose, content, and method of the study to all the participants or substitutes, and that the data would be analyzed at Okayama Prefectural University. The researchers also explained the respect for the rights of the individual and guarantee of voluntariness (interruption is possible) of cooperation, that there would be no disadvantages due to non-participation or withdrawal, the anonymity of all obtained data (statistically processed without identifying information), and disclosure. The person (or substitute) signed the research agreement and obtained consent. The researchers also explained that consent could be withdrawn even after sign in, and issued a consent withdrawal letter. Before starting the study, the protocol was approved by the Research Ethics Committee of Okayama Prefectural University (Approval No. 18-70) and the National Institute of Biomedical Innovation (Approval No. 219-01) in accordance with the Declaration of Helsinki. This study is registered in the clinical trial registration system (UMIN000043009).

### 2.2. Attributes and Physical Condition

Sex, age, and blood pressure were obtained from medical records. For height, weight, and BMI, the most recent measurements were obtained from facility records. We used the Clinical Frailty Scale (CFS), which was created based on the research report of Rockwood et al. [10]. The facility staff responded. Grip strength was measured with a grip strength meter, the GRI-A (Takei Scientific Instruments Co., Ltd., Niigata, Japan), to evaluate muscle strength (one participant with paralysis was excluded). We measured the subjects’ grip strength according to the “Grip Strength Measurement of the Elderly” reported by the Japan Foundation for Aging and Health. Body composition was measured in the supine position using an MLT-550N (Toray Medical Co., Ltd., Tokyo, Japan) with the multi-frequency bioelectrical impedance method.

### 2.3. Blood Test

Fourteen blood test parameters were investigated: white blood cell count, red blood cell count, hemoglobin, hematocrit, platelet count, mean corpuscular volume (MCV), mean corpuscular hemoglobin (MCH), mean corpuscular hemoglobin concentration (MCHC), basophils, eosinophils, neutrophils, lymphocytes, monospheres, and total lymphocytes. Nineteen biochemical test parameters were investigated: cholinesterase, total protein, albumin, albumin/globulin ratio, total cholesterol, neutral fat, HDL cholesterol (HDL-C), LDL cholesterol (LDL-C), atherosclerosis index, urea nitrogen (BUN), creatinine, estimated glomerular filtration rate (eGFR (creatinine)), hemoglobin A1c (HbA1c), C-reactive protein (CRP), total carnitine, free carnitine, acyl carnitine, trimethylamine N-oxide (TMAO), and N-terminal pro-brain natriuretic peptide (NT-proBNP). The quantitative analysis was outsourced.

### 2.4. Mini Mental State Examination (MMSE)

The MMSE, which evaluates orientation, memorization, attention and calculation, reproduction, naming, recitation, comprehension, reading, writing, and drawing based on 11 question items, was used to evaluate the cognitive function. The MMSE was administered by care staff. The questionnaire has a maximum score of 30 points, with the cut-off score set at 23 points for older people in Japan, and has demonstrated sensitivity and specificity for identifying cognitive impairment in older people [11].

### 2.5. Nutritional Status

Nutritional intake was assessed by measuring the nutritional composition of all meals provided at the facility over a 7-day period using Excel Eiyo-kun (Science Forum Co., Ltd., Gifu Prefecture, Japan). For individuals receiving oral nutrition, the average amount of nutrients provided during the most recent week was multiplied by the individual intake rates of staple foods and other foods during that period to estimate the actual nutritional intake per person. For those receiving tube feeding, nutritional intake was calculated based on the types of nutritional products administered during each period, along with the nutritional composition and individual intake rates disclosed by each manufacturer. Nutritional supplements are also included in the calculations. All values related to the amounts of nutrients provided and nutritional intake were verified by the facility’s registered dietitian.

We used the Mini Nutritional Assessment (MNA) to assess nutritional status. The MNA is recommended for the screening of older people in the 2002 guidelines of the European Society for Intravenous and Enteral Nutrition (ESPEN) [12]. The MNA is used to detect undernutrition and the risk of developing undernutrition among older individuals in home-care programs, nursing homes, and hospitals. This questionnaire evaluates 18 items, including screening items and assessment items, on a scale of 30 points [13]. Facility staff answered for participants who had difficulty communicating.

### 2.6. Cardiac Function

Echocardiography was conducted by a medical doctor who is an investigator to assess left ventricular ejection fraction (LVEF), inferior vena cava minimum diameter (IVCins), inferior vena cava maximum diameter (IVCexp), and degree of valvular heart disease.

### 2.7. Authorized Questionnaire

We used an authorized questionnaire [14] for obstacle-degree division authorization in certification for long-term care. We checked items about body function and movement behavior, life function, cognitive function, mind, behavior disorders, and adaptation to social life among authorized questionnaires. The respondent was a staff member of one of the facilities. The total score was 170.

### 2.8. Cohen-Mansfield Agitation Inventory (CMAI)

The CMAI is a scale for caregivers to assess behavioral symptoms in older individuals with dementia [15]. We used it to evaluate the frequency of specific behavioral disorders over a period of time. Answers were given by facility staff.

### 2.9. Behavioral Ability

We created a questionnaire about behavioral ability. The items were “1: Bedridden”; “2: Can stand up”; “3: Can walk indoors”; “4: Can walk in the corridor or grab and walk”; “5: Can walk up and down stairs”; “6: Can walk (<2000 steps/day)”; and “7: No inconvenience (≥2000 steps/day)”. Scores ranged from 1 to 7 points. Answers were given by facility staff.

### 2.10. Bowel Movement Situation Investigation

We investigated the number of independent excretions, excretions on pads, and diarrhea episodes from the medical record.

We used the Japanese version of the Constipation Evaluation Scale (CAS) to evaluate constipation symptoms. The mid-term CAS questionnaire, which evaluates the past week, was used. It consists of eight items related to constipation symptoms [16]. Subjective symptoms were evaluated, with a total score of up to 16 points. The stronger the tendency for constipation, the higher the CAS score. Constipation is defined as a total score of ≥5 points.

The Bristol Scale (BSS) was used to determine the stool condition. This stool property score visually classifies the stool shape and hardness into seven levels, from hard to loose stool [17], reflecting the transit time in the intestinal tract. Its validity has been clarified as a subjective evaluation method for constipation and diarrhea [18]. Respondents and caregivers evaluated participants’ stools. These evaluators remained the same during the survey period. Based on previous studies [19], the participants’ stool properties were evaluated.

Due to difficulties in self-evaluation of the BSS and CAS themselves, responses were provided by caregivers. The frequency of defecation, enema use, and laxative use was investigated from medical records.

### 2.11. Gut Microbiome

#### 2.11.1. Sample Collection

Fecal samples were collected from 21 individuals in guanidine thiocyanate solution (TechnoSuruga Laboratory, Shizuoka, Japan). These samples were stored at 4 °C and processed for DNA extraction within one week of collection, specifically for 16S rRNA gene amplicon sequencing.

#### 2.11.2. DNA Extraction

DNA was extracted from the fecal sample mixture using an automatic nucleic acid extractor (Kurabo Industries Ltd., Osaka, Japan) by partially modifying the previously reported protocol [20]. Homogenization of the stool samples (rice-grain-sized) was performed using beads with 500 µL of lysis buffer (No. 10, Kurabo Industries Ltd., Osaka, Japan) and 0.5 g of 0.1 mm glass beads in 2 mL vials. The mixture was mechanically disrupted using a Cell destroyer PS1000 (Bio-Medical Science, Tokyo, Japan) at 4260 rpm for 50 s at room temperature (20–25 °C). All samples were centrifuged at 12,000× *g* for 5 min at room temperature. A total of 200 µL of supernatant was collected and mixed with 150 µL of lysis buffer and 150 µL of proteinase K buffer (No. 2, Kurabo Industries Ltd., Osaka, Japan) containing 0.4 mg/mL of proteinase K. DNA was extracted using a Gene Prep Star PI-80X (Kurabo Industries Ltd., Osaka, Japan). Extracted DNA was analyzed using a NanoDrop Spectrophotometer ND-1000 (Thermo Fisher Scientific Inc., Waltham, MA, USA), and samples were stored at −30 °C.

#### 2.11.3. 16S rRNA Sequencing

16S rRNA sequencing was performed as described previously [20]. The V3-V4 region of the 16S rRNA gene was amplified from salivary DNA samples using previously published primers. The reaction process was performed at 95 °C for 3 min, followed by 25 cycles at 95 °C for 30 s, 55 °C for 30 s, and 68 °C for 1 min, with a final extension at 68 °C for 5 min. PCR products were purified with Agencourt AMPure XP (Beckman Coulter, Inc., Brea, CA, USA) in accordance with the manufacturer’s protocol and eluted into 50 µL of 10 mM Tris-HCl, pH 8.5. For DNA library preparation, Illumina adapters were attached to PCR products using an Illumina MiSeq Nextera kit set A (Illumina Inc., San Diego, CA, USA). 16S rRNA gene sequencing of PCR products was performed using an Illumina MiSeq (Illumina, San Diego, CA, USA) according to the manufacturer’s instructions. Ten thousand reads per sample were randomly selected for a further analysis. Samples with insufficient read numbers were re-sequenced, and samples with repeated insufficient read numbers were thereafter excluded.

#### 2.11.4. Bioinformatics Analysis

FASTQ files were obtained after Illumina pair-end 16S rRNA gene amplicon sequencing, and operational taxonomic unit (OTU) classification and diversity analyses were performed using QIIME version 1.9.1 [21] according to previously described methods [22]. Sequences were clustered into OTUs by an open-reference OTU picking process against the SILVA 128 reference database [23] at 97% similarity using USEARCH [24]. OTUs were classified taxonomically up to the genus level using the SILVA 128 reference database [23].

### 2.12. Statistical Analysis

Resulting data were exported as BIOM files and imported to R (version 3.5.0). A diversity analysis was performed using the “phyloseq” R package [25]. The alpha-diversity indexes (OTU observed, Chao 1 Index, Shannon Index, and Simpson Index) were calculated by the estimate_richness function. The beta-diversity index, calculated by the Bray–Curtis distance using genus-level data, was generated using the vegdist function in the “vegan” R package. Gut microbiome community structure similarity of each sample was calculated using principal coordinate analysis (dudi. pco function in “ade4” R package). The dominant bacteria from phylum to genus level were defined as at least 1% of the mean of the distribution of bacterial composition. We conducted analyses specifically at the phylum, family, and genus levels to ensure a comprehensive understanding of the bacterial communities present.

In addition, we conducted a clustering analysis to identify distinct subgroups among participants based on 239 clinical assessment items. Data for these items were collected from older residents in nursing homes, with each participant undergoing a comprehensive evaluation that included attributes such as physical condition, blood tests, the Mini Mental State Examination (MMSE), nutritional status, cardiac function, and behavioral assessments, including the Cohen-Mansfield Agitation Inventory (CMAI). Prior to clustering, we performed data preprocessing, including normalization and handling of missing values to ensure that all clinical items were on a comparable scale.

Hierarchical clustering was utilized for the dataset, informed by the nature of the continuous variables. We initially performed principal component analysis to reduce data dimensions, followed by hierarchical clustering using Ward’s criteria. The final clusters were refined through k-means clustering methods. The Kruskal–Wallis test was employed to compare the three groups using the Statistics Premium Grad Pack Version 26 (IBM, Armonk, NY, USA). All statistical analyses were performed using R (version 3.5.0), with a significance level set at *p* < 0.05.

## 3. Results

### 3.1. Characteristics of Older People Living in an Institution Based on Clinical Phenotypes

To understand the various clinical phenotypes of frail older people, we focused on individuals aged 65 years or older residing in a single nursing home. We conducted a cross-sectional study involving 21 older people who exhibited high frailty scale scores. The frailty scale score was the average of 6.9 ± 0.3. Eighteen participants had a score of 7 (“Requires assistance in all aspects of life, both physical and cognitive. However, the physical condition is stable, and the risk of death [within half a year] is not high”). Three participants had a score of 6 (“Support is required for all outdoor activities and housework. It becomes difficult to go up and down stairs, and assistance is required for bathing. In some cases, support for changing clothes is required”). Accordingly, all participants had a very high degree of frailty.

The average age was 86.5 ± 8.5 years, and 14 participants were the oldest-old (aged 85 and older). The maximum and minimum MMSE values were 22 and 0 points, respectively, suggesting that all participants had cognitive impairment because a score of ≤23 indicates suspected dementia. The average BMI was 20.0 ± 4.3 kg/m^2^, which is slightly lower than the target value of 21.5–24.9 kg/m^2^ according to the Dietary Reference Intakes for Japanese (2020) to prevent frailty and the onset of lifestyle-related diseases. In this study, participants were primarily older adults meeting nursing home admission criteria, specifically those requiring a high level of nursing care categorized as care level ≥3.

### 3.2. Grouping of Participants Based on Hierarchical Clustering on Principal Components (HCPC)

**Figure 1:**
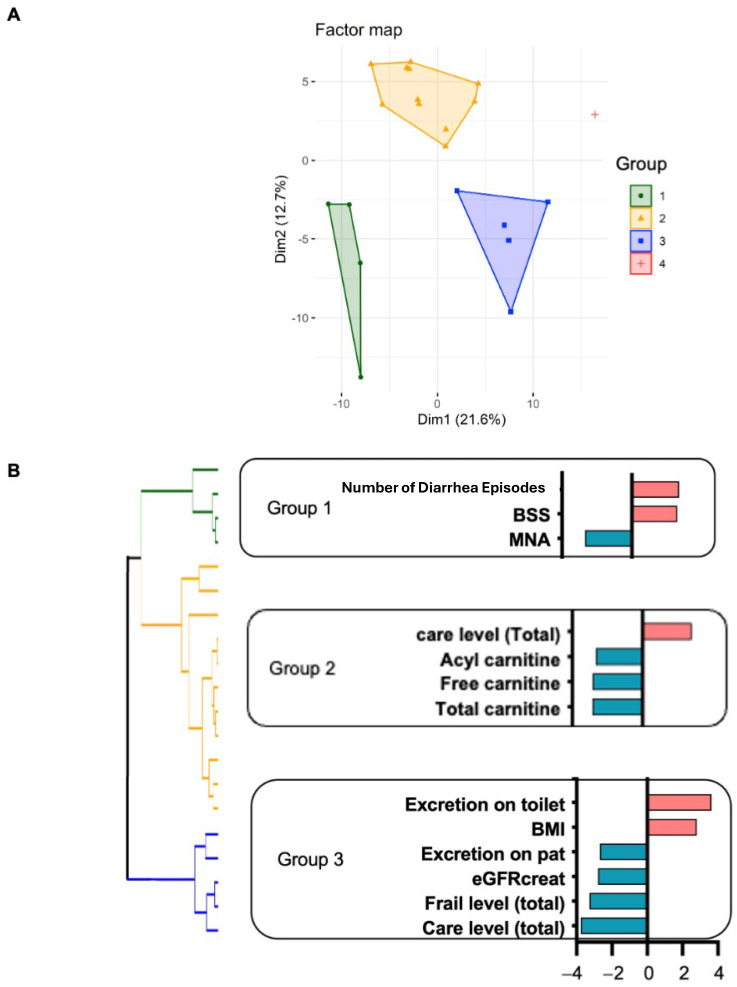
The clinical phenotypes of frail older individuals. (**A**) Principal component analysis (PCA): The PCA factor map shows four distinct clusters based on participant characteristics. Cluster 1 (green circles) represents similar traits, Cluster 2 (orange triangles) indicates a different subgroup, Cluster 3 (blue squares) highlights another unique group, and Cluster 4 (pink diamonds) depicts a separate cluster. Dim1 accounts for 21.6% of the variance, while Dim2 accounts for 12.7%. Each point corresponds to an individual clustered by analyzed features. (**B**) Hierarchical clustering on principal components (HCPC) and a comparative analysis: characterization the clinical phenotype in each group; we listed the items with *p* < 0.01 in a comparative analysis, excluding the questions in each survey and various nutrients.

To understand the various clinical phenotypes in frail older individuals, we performed HCPC using clinical metadata, including dietary habits and nutrition, blood data, nursing care information, defecation situation, cognitive function, and behavioral disorder status. A principal component analysis (PCA) and HCPC dendrogram showed that frail older people could be divided into four groups (Figure 1A,B): groups 1 (*n* = 4), 2 (*n* = 11), 3 (*n* = 5), and 4 (*n* = 1). Group 4 (*n* = 1) was excluded due to the small sample size, and the analysis was conducted on the remaining three groups. These results suggest that older institutional residents had the same degree of frailty but various clinical phenotypes and that they can be divided into at least three categories.

### 3.3. Characteristic Clinical Phenotypes of Each Group

To reveal the characteristics of the clinical phenotype in each group, the clinical metadata of 239 evaluated items were compared among the groups. Among eight comprehensive evaluation items and four blood data items, in group 1, the total diarrhea episodes (*p* = 0.0068) and BSS (*p* = 0.0093) were significantly higher, and the overall MNA evaluation value (*p* = 0.0074) was significantly lower than in the other groups (Figure 1B). The average BMI was 17 ± 2.6 kg/m^2^, the GNRI was 82 ± 5.5 (moderate nutritional risk), and the MNA results showed that group 1 had the worst nutritional status. Accordingly, group 1 (*n* = 4) was characterized by loose stool, frequent diarrhea, and a poor nutritional status in comparison to groups 2 and 3.

Group 2 had the largest number of people (*n* = 11). The average age was 85 ± 8.3 years (the youngest group), but it was characterized by a significantly higher total care level score (*p* = 0.0052), indicating a more serious long-term care state (Figure 1B). The nutritional status, based on MNA and GNRI (84 ± 7.6), was better than in group 1, but the nutritional risk was moderate. Total carnitine (*p* = 0.0043) and free carnitine (*p* = 0.0045) levels were significantly lower in group 2 (Figure 1B). However, no subjects had free carnitine <20 μmol/L (diagnostic criterion for carnitine deficiency) or 20–36 μmol/L with an acylcarnitine-to-free-carnitine ratio of >0.4 (indicating an extremely high likelihood of developing deficiency). Serum carnitine levels are essential for mitochondrial energy production and are abundant in skeletal muscle. Therefore, the high care level score suggests a negative impact on energy production and a loss of skeletal muscle. Additionally, carnitine may be involved in muscle atrophy associated with aging, as well as the effect of improved nutritional status on energy production. Future research should further explore the effects of carnitine supplementation on muscle function and overall health in older adults. 

The average age of group 3 was 93 ± 4.8 years (the oldest group), but the average BMI was 25 ± 4.5 kg/m^2^, which exceeded the target BMI for older individuals. The nutritional status of group 3 was also the best among the three groups based on MNA and GNRI (94 ± 3.9). In addition, this group had significantly better values for toilet excretion (number of times they could go to the toilet by themselves) (*p* = 0.0003) and BMI (*p* = 0.0049), pad excretion (*p* = 0.0064), frailty evaluation score (*p* = 0.0011), and total care level (*p* = 0.0002). From these results, five people in group 3 had the highest degree of independence. In blood data, lower eGFR (creatinine) (*p* = 0.0052) suggested renal dysfunction. However, we should carefully comprehend this result because blood eGFR (creatinine) may be overestimated independently of renal function in older people with low muscle mass [26]. The results showed that there was a significant difference in calf circumference (CC) among the three groups (*p* = 0.014), which is known to correlate well with limb skeletal muscle mass, and that group 3 had a significantly longer CC than group 1. Additionally, a strong correlation was observed between CC and eGFR (creatinine) (*p* < 0.001), indicating that muscle mass has a significant effect on the apparent “index of the renal function”.

### 3.4. Clinical Phenotype

**Figure 2:**
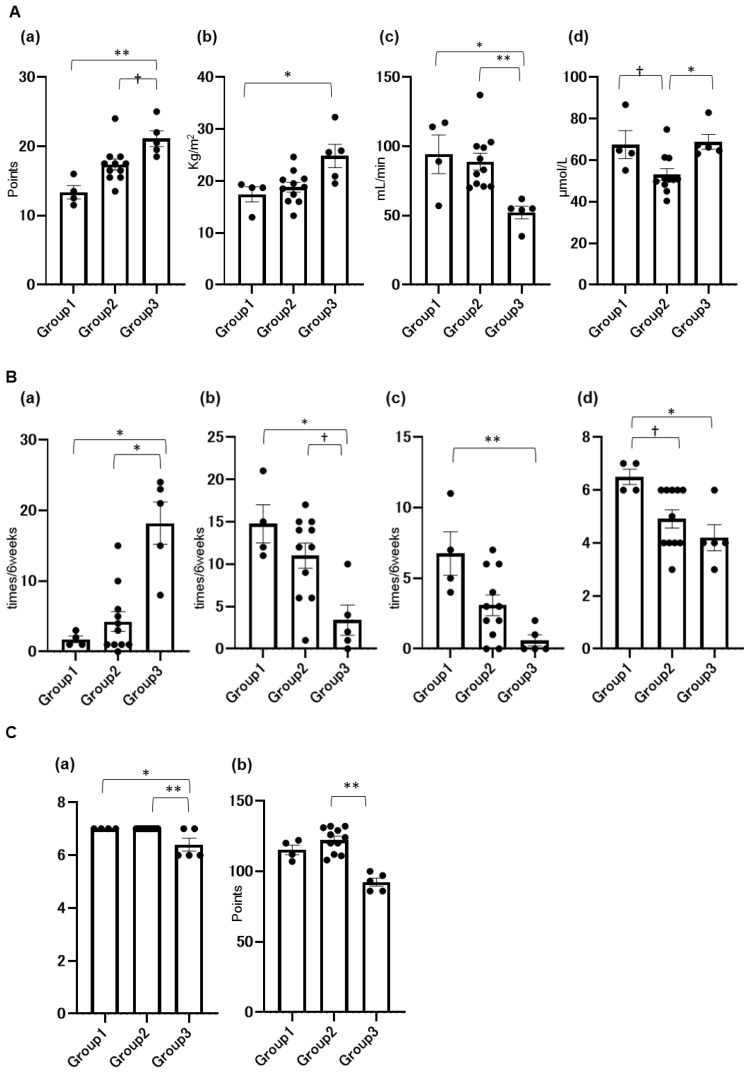
Intergroup comparison of items characterizing the clinical phenotype of each group. (**A**) Nutritional status: (a) MNA, (b) BMI, (c) eGFR (creatinine), (d) total carnitine. (**B**) Defecation: (a) toilet defecation, (b) excretions on pad, (c) diarrhea, (d) BSS. (**C**) Care needs: (a) Clinical Frailty Scale (CFS), (b) care level (point). Data are shown as the mean ± SEM. Comparison between groups: Mann–Whitney U test. ** *p* < 0.01, * *p* < 0.05, ^†^ *p* < 0.1.

Figure 2A–C show the ten items that characterize the clinical phenotype of each group by comparative analysis. Items characterizing nutritional status are the MNA comprehensive evaluation value, BMI, and total carnitine in serum (Figure 2A). Items characterizing defecation are the total toilet excretions, total excretions on pada, total diarrhea episodes, and BSS (Figure 2B). Items characterizing care needs are the total score of frailty evaluation and care level (Figure 2C).

The clinical phenotype of each group is summarized as follows: group 1, dementia, malnutrition, tube feeding, and poor defecation status; group 2, dementia, undernutrition, and low carnitine; group 3, dementia alone with a better nutritional and defecation status.

### 3.5. Correlation of Items Characterizing the Clinical Phenotype

**Figure 3:**
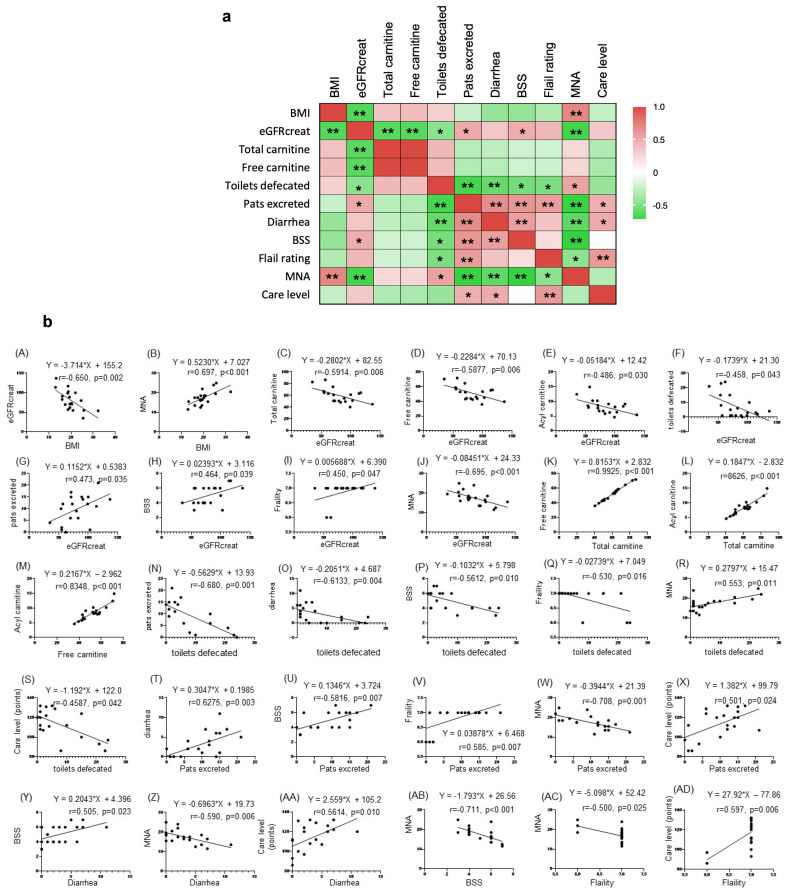

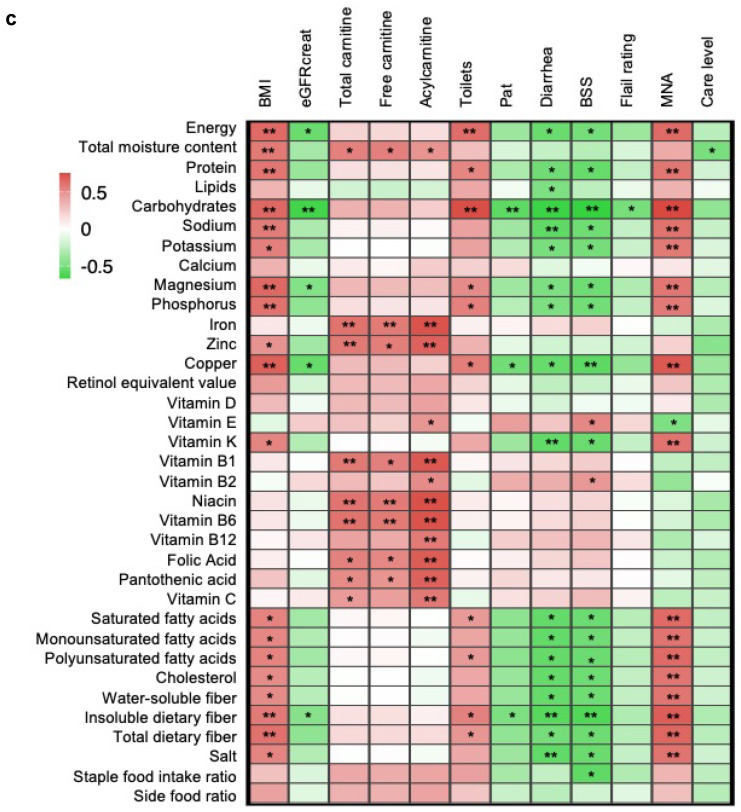
Correlation matrix heatmap between items characterizing the clinical phenotype of each group, as determined by a comparative analysis. (**a**) Correlation analysis (heatmap): Spearman’s correlation: ** *p* < 0.01, * *p* < 0.05. (**b**) Correlation analysis (plot diagram): plot diagrams are shown for items with *p* < 0.05 according to Spearman’s correlation. (A) eGFRcreat vs. BMI (*p* = 0.002) (B) BMI vs. MNA (*p* < 0.001) (C) eGFRcreat vs. total carnitine (*p* = 0.006) (D) eGFRcreat vs. free carnitine (*p* = 0.006) (E) eGFRcreat vs. acyl carnitine (*p* = 0.030) (F) eGFRcreat vs. toilets detected (*p* = 0.043) (G) eGFRcreat vs. pats excreted (*p* = 0.035) (H) eGFRcreat vs. BSS (*p* = 0.039) (I) eGFRcreat vs. frailty (*p* = 0.047) (J) eGFRcreat vs. MNA (*p* < 0.001) (K) free carnitine vs. acyl carnitine (*p* < 0.001) (L) total carnitine vs. acyl carnitine (*p* < 0.001) (M) free carnitine vs. pats excreted (*p* < 0.001) (N) toilets defecated vs. pats excreted (*p* = 0.016) (O) toilets defecated vs. diarrhea (*p* = 0.004) (P) toilets defecated vs. BSS (*p* = 0.010) (Q) toilets defecated vs. frailty (*p* = 0.016) (R) toilets defecated vs. MNA (*p* = 0.011) (S) Care level vs. toilets defeated (*p* = 0.042) (T) Diarrhea vs. pats excreted (*p* = 0.003) (U) BSS vs. pats excreted (*p* = 0.007) (V) Frailty vs. pats excreted (*p* = 0.007) (W) MNA vs. pats excreted (*p* = 0.001) (X) Care level vs. pats excreted (*p* = 0.024) (Y) BSS vs. diarrhea (*p* = 0.023) (Z) MNA vs. diarrhea (*p* = 0.006) (AA) Care level vs. diarrhea (*p* = 0.010) (AB) MNA vs. BSS (*p* < 0.001) (AC) MNA vs. frailty (*p* = 0.025) (AD) Care level vs. frailty (*p* = 0.006). (**c**) Correlation matrix heatmap between nutrients and items characterizing the clinical phenotype. Spearman’s correlation: ** *p* < 0.01, * *p* < 0.05.

To determine whether other clinical factors were affected by the clinical characteristics of each group, we examined the correlation of data in each group. Among the parameters characterizing group 1, defecation parameters, including the total diarrhea episodes and BSS, were negatively correlated with MNA (Spearman’s correlation −0.590 and −0.711, respectively) (Figure 3A,B and Figure S1), suggesting that the higher the frequency of diarrhea and soft stools, the worse the nutritional status. Additionally, defecation parameters were positively correlated with excretions on pads. MNA nutrition was positively correlated with BMI and toilet defecation but negatively correlated with frailty rating and excretions on pads (Figure 3A,B and Figure S1). Collectively, we hypothesize that deterioration in the eating state and defecation condition lead to nursing care requirements due the higher need for toilet care and a decline in physical fitness, as estimated by decreased BMI.

As parameters directly correlated with the care level, a characteristic of group 2, the numbers of excretions on pads and diarrhea episodes and the BSS showed *p* values by Spearman’s correlation of *p* = 0.024, *p* = 0.010, and *p* = 0.005, respectively (Figure 3A,B and Figure S1), suggesting that the higher the number of excretions on pads, diarrhea episodes, and soft stool episodes, the worse the care status.

The better BMI and nutritional status were considered to explain why people in group 3 could live more independently than the other groups.

### 3.6. Correlation Between Items That Characterize the Clinical Phenotype and Nutrient Intake

To examine appropriate dietary care for each group, we investigated the nutrients associated with the items that characterized the clinical phenotype of each group. A heatmap (Figure 3C and Supplementary Materials Figures S2 and S3) shows the correlation between nutrients and the 12 items that characterize the clinical phenotype.

Items that were positively correlated with the 35 nutrients were BMI, total carnitine, total toilet excretions, and MNA, suggesting that as nutrient intake increases, the number of trips to the toilet increases, leading to a better nutritional status and an increased BMI. In contrast, eGFR (creatinine), total excretions on pads, total diarrhea episodes, BSS, frailty rating, and care level had a negative correlation with nutrients, suggesting that the *increased nutrient intake may reduce the frequency of diarrhea and soft stools and improve caregiving status and frailty ratings.*

### 3.7. Relationship Between Clinical Phenotype and Gut Microbiome

**Figure 4:**
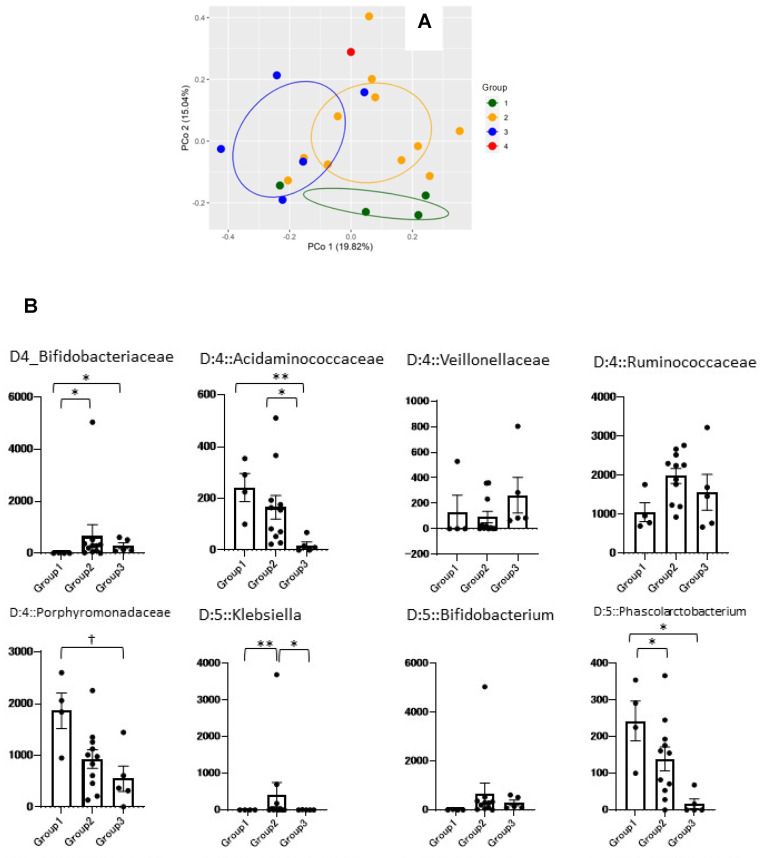

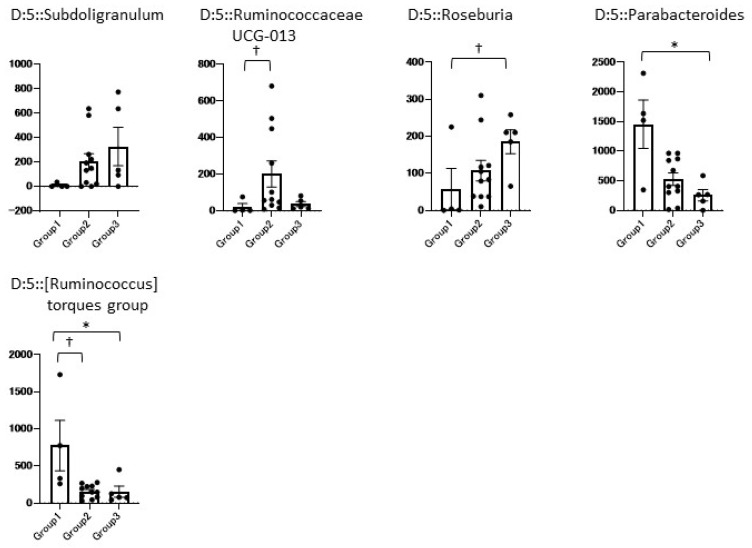
Characteristics of the intestinal microbiota of each group with different clinical phenotypes. (**A**) Principal coordinate analysis (PCoA) of the intestinal microbiota at the genus level. (**B**) Group comparison at the genus (D:5) or family (D:4) level. Data are shown as the mean ± SEM. Comparison between groups was performed using the Mann–Whitney U test; ** *p* < 0.01, * *p* < 0.05, ^†^ *p* < 0.1.

To investigate possible differences in the gut microbiome of four groups with different clinical phenotypes, a principal coordinate analysis was performed on the genus level of the gut microbiome, which identified a significant difference among the four groups (*p* = 0.007) (Figure 4A). This revealed that the gut microbiome differed in subgroups with the same degree of frailty but different clinical phenotypes.

To compare the gut microbiome of the groups, we also examined differences at the family and genus levels. Significant differences were observed in the five family levels (*Bifidobacteriaceae*, *Acidaminococcaceae*, *Veillonellaceae*, *Ruminococcaceae*, *Porphyromonadaceae*) and eight genus levels (*Klebsiella*, *Bifidobacterium*, *Phascolarctobacterium*, *Subdoligranulum*, *Ruminococcaceae UCG-013*, *Roseburia*, *Parabacteroides*, *[Ruminococcus]torques group*) (Figure 4B).

### 3.8. Correlation Between Items Characterizing the Clinical Phenotype and Gut Microbiome

**Figure 5:**
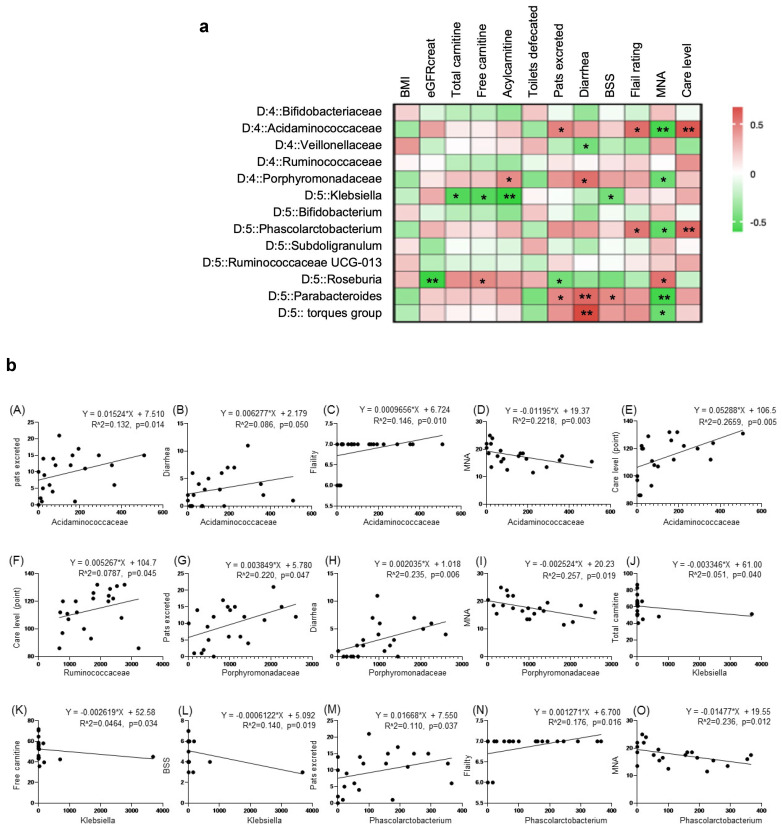

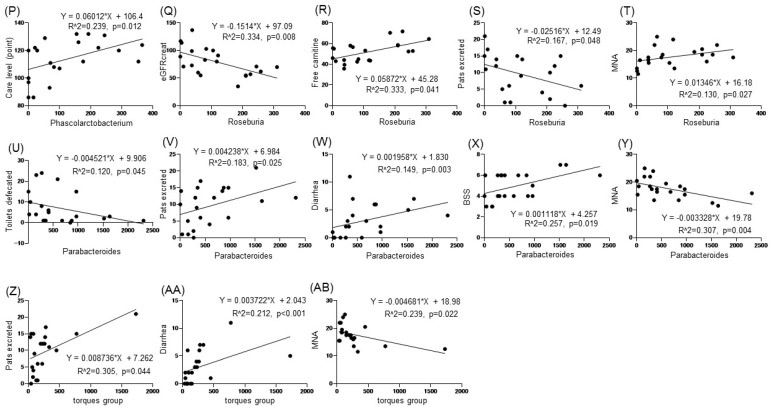
Correlation analysis between gut microbiome and items characterizing the clinical phenotype. (**a**) Correlation analysis (heatmap): Spearman’s correlation: ** *p* < 0.01, * *p* < 0.05. (**b**) Correlation analysis (plot diagram): plot diagrams are shown for items with *p* < 0.05 according to Spearman’s correlation. (A) *Acidaminococcaceae* vs. pats excreted (*p* = 0.014) (B) *Acidaminococcaceae* vs. diarrhea (*p* = 0.050) (C) *Acidaminococcaceae* vs. frailty (*p* = 0.010) (D) *Acidaminococcaceae* vs. MNA (*p* = 0.003) (E) *Acidaminococcaceae* vs. care level (*p* = 0.005) (F) *Ruminococcaceae* vs. care level (*p* = 0.045) (G) *Porphyromonadaceae* vs. pats excreted (*p* = 0.047) (H) *Porphyromonadaceae* vs. diarrhea (*p* = 0.006) (I) *Porphyromonadaceae* vs. MNA (*p* = 0.019) (J) *Klebsiella* vs. total carnitine (*p* = 0.040) (K) *Klebsiella* vs. free carnitine (*p* = 0.034) (L) *Klebsiella* vs. BSS (*p* = 0.019) (M) *Phascolarctobacterium* vs. pats excreted (*p* = 0.037) (N) *Phascolarctobacterium* vs. frailty (*p* = 0.012) (O) *Phascolarctobacterium* vs. MNA (*p* = 0.016). (P) *Phascolarctobacterium* vs. care level (*p* = 0.012) (Q) *Roseburia* vs. eGFR (*p* = 0.008) (R) *Roseburia* vs. free carnitine (*p* = 0.041) (S) *Roseburia* vs. pats excreted (*p* = 0.048) (T) *Roseburia* vs. MNA (*p* = 0.027) (U) *Parabacteriodes* vs. toilets detected (*p* = 0.045) (V) *Parabacteriodes* vs. pats excreted (*p* = 0.025) (W) *Parabacteriodes* vs. diarrhea (*p* = 0.003) (X) *Parabacteriodes* vs. BSS (*p* = 0.019) (Y) *Parabacteriodes* vs. MNA (*p* = 0.004) (Z) *torques group* vs. pats excreted (*p* = 0.044) (AA) *torques group* vs. diarrhea (*p* < 0.001) (AB) *torques group* vs. MNA (*p* = 0.022); Spearman’s correlation: ** *p* < 0.01, * *p* < 0.05.

To determine whether the gut microbiome influenced the clinical characteristics of each group, we investigated the association between the gut microbiome and items characterizing the clinical phenotype of each group. The heatmap in Figure 5 shows that the correlation between the ten items characterizing the clinical phenotype and genus-level gut microbiome (13 types) differed between groups. *Klebsiella* was significantly correlated with total carnitine (r = −0.547) and BSS (r = −0.457). *Phascolarctobacterium* was significantly correlated with frailty rating (r = 0.524), care level (r = 0.569), and MNA (r = −0.508). Parabacteroides was negatively correlated with total excretions on pads (r = 0.466), total diarrhea episodes (r = 0.575), BSS (r = 0.458), and MNA (r = −0.584). The *[Ruminococcus] torques* group was significantly correlated with total diarrhea episodes (r = 0.680) and MNA (r = −0.481). *Roseburia* was significantly correlated with MNA (r = 0.538) and total excretions on pads (r = −0.458). No correlation was found with *Subdoligranulum*, *Bifidobacterium*, or *Ruminococcaceae UCG-013*.

## 4. Discussion

We investigated the clinical phenotype of frailty and its association with the gut microbiome in frail older Japanese individuals in a nursing care setting. Using principal component analysis (PCA) for clustering, we identified four groups with distinct clinical phenotypes, characterized by parameters including degree of frailty, nutritional status, defecation status, and nursing care dependency. The relationships with the gut microbiome were examined in three groups that displayed characteristic clinical phenotypes, revealing statistically significant differences. These findings indicate that gut microbiome composition varies according to clinical frailty phenotypes and supports numerous reports that highlight the involvement of the gut microbiome in various diseases and physical conditions. However, whether the observed differences in the gut microbiome are a cause or consequence of the phenotypic variations remains to be determined in future studies. The results of this study made it clear that malnutrition is the most important issue in clinical management at elderly care facilities. Specific measures that are important for preventing malnutrition and the need for nursing care include maintaining bowel independence, ensuring sufficient food intake, and incorporating a diet that takes into account the intestinal flora. By focusing on these points, and with the efforts of both the elderly people themselves and the nursing staff, it is believed that the chances of elderly people living with dignity will be greatly increased.

Currently, frailty is conceptualized primarily through two models: the phenotype model and the deficit-accumulation concept. While the phenotype model, as established by Fried et al. [4], is useful for assessing individuals in pre-care settings, it is not well suited for older adults requiring nursing care [27]. Therefore, given that our study participants are elderly individuals in nursing care settings, we concentrated on the deficit-accumulation model, which is more appropriate for understanding frailty in its broader context. We employed the Clinical Frailty Scale (CFS) [28], derived from this model, to evaluate dependency, physical function, and cognitive ability. The majority of participants (85%) were classified as CFS grade 7. Clustering based on 239 parameters led to the identification of four distinct groups; however, one group was excluded due to a statistically insufficient sample size.

Group 1 consisted entirely of enterally fed individuals, characterized by the poorest nutritional status (BMI: 17 ± 2.6 kg/m^2^, GNRI: 82 ± 5.5) and frequent diarrhea. Group 2, the youngest cohort (mean age: 85 ± 8.3 years), exhibited the highest care dependency and low serum carnitine levels, suggesting impaired energy production. Group 3 was the oldest (mean age: 93 ± 4.8 years) and showed the best nutritional status (BMI: 25 ± 4.5 kg/m^2^, GNRI: 94 ± 3.9), along with greater independence in bowel management, which likely contributed to an enhanced quality of life.

Malnutrition is prevalent among older adults and often challenging to treat, even with nutritional therapy. Recent studies indicate that alterations in the gut microbiome contribute to persistent malnutrition by affecting intestinal permeability and nutrient absorption [8]. Similar changes have been observed in children with kwashiorkor, where gut microbiome differences persisted despite dietary improvements [8]. Furthermore, imbalances in gut microbiota have been linked to reduced production of short-chain fatty acids (SCFAs) and other metabolites essential for maintaining intestinal health, exacerbating nutritional deficiencies [29].

Regarding frailty and the gut microbiome, decreases in *Eubacterium*, *Faecalibacterium*, and *Lactobacillus* populations [30], alongside increases in *Prevotella*, have been documented [31,32]. *Eubacterium*, in particular, produces SCFAs (especially butyric acid) [33], which modulate immune responses [34] and are considered protective for colon health. Our findings reveal a reduction in *Eubacterium* among older individuals with frailty, supporting the notion of SCFAs’ protective role in gut health. However, the precise mechanisms underlying these observations remain unclear.

The results of this study suggest that “low food intake” and “the need for meal assistance due to cognitive decline” are closely related to nutritional status. Notably, all participants in group 1 were receiving enteral nutrition. The differences in gut microbiome composition among the various clinical phenotypes identified through PCA may largely result from variations in nutritional and defecation statuses. While this possibility warrants thorough investigation, interventions aimed at modulating the gut microbiome could significantly enhance the quality of life in older adults. Notably, within the three groups exhibiting distinct clinical phenotypes, both nutritional status and gut microbiome composition varied significantly, suggesting that nutritional status plays a central role in determining clinical phenotype. This underscores the importance of maintaining nutritional status for independent living among older adults, which may be more critical than previously thought. However, longitudinal or interventional studies are necessary to establish whether modulating the gut microbiome (e.g., through diet, probiotics, or fecal microbiota transplantation) can indeed alter the trajectory of frailty in older adults.

Prior studies comparing frail and non-frail groups may have overlooked the clinical phenotypic distinctions identified in our research. To explore the association between frailty severity and the gut microbiome, future investigations should include older adults across a broader range of frailty levels. Although no significant differences were found in *Bifidobacterium* levels (linked to constipation), notable variations emerged at the family level for *Bifidobacteriaceae*, highlighting the need for cautious interpretation, particularly as no correlation with diarrhea frequency or stool consistency was observed.

Pad defecation frequency, CFS, and care dependency were positively correlated with the abundance of *Phascolarctobacterium* and *Acidaminococcaceae*. *Acidaminococcus*, which relies on amino acids for growth, may reflect amino acid malabsorption and could relate to serum protein loss in frail older adults. An increase in *Phascolarctobacterium* has been documented in early-stage colorectal cancer (CRC) patients, where elevated levels of succinate and lactate in fecal metabolites have been observed [35,36]. Further research is necessary to elucidate the roles of these metabolites in frailty.

## 5. Limitation

The sample size in this study was relatively small (*n* = 21), necessitating further research with a larger cohort to draw reliable and generalizable conclusions about whether there is any bias in the identified clinical phenotypes of frailty. We acknowledge that the results of this study do not indicate causation and may only be correlational. This study adopts an approach to identify clinical phenotypes from a single facility. As a result, the study design may not fully capture the multifaceted nature of frailty in the broader population. In particular, clinical phenotypes related to cardiac dysfunction, which are frequently seen in older populations, should be investigated by including patients with heart disease. This would allow for an examination of the potential impact of cardiac function on frailty. Additionally, the study population consisted of 21 institutionalized older adults, over 85% of whom were classified as CFS grade 7. To gain a comprehensive understanding of frailty phenotypes, future research should include older adults across a broader spectrum of CFS grades and replicate similar analyses. As metagenomic analysis methods are evolving rapidly, it would be desirable to use the latest techniques, such as information analysis using QIIME 2 and shotgun sequencing. However, in this study, we adopted the analysis method using 16S and QIIME 1 due to budget and equipment constraints. We believe that validating our findings with the latest methods in the future would be beneficial.

## 6. Conclusions

In this study, participants were classified into subgroups using clustering analysis with 239 clinical survey items, and group characteristics were compared. Each group exhibited distinct clinical phenotypes based on nutritional status, bowel habits, and caregiving needs. Notably, malnutrition emerged as a key factor in differentiating the core clinical phenotypes. Furthermore, the gut microbiota composition differed significantly between the groups (*p* = 0.007), showing a correlation with changes in clinical phenotypes.

## Figures and Tables

**Table 1:** Participant characteristics.

No.	Age	^1^ BMI (kg/m^2^)	Frailty Score	^2^ MMSE	^3^ GNRI	^4^ AC (cm)	^5^ CC (cm)	Energy Intake (kcal)	Intake Method	NT-proBNP (pg/mL)	^6^ CAS	^7^ BSS	HCPC Group
1	68	20.4	7	0	93.5	26.1	26.4	1332	Oral	53.6	8	4	2
2	70	19.6	7	12	91.2	24.0	28.9	1533	Oral	677	6	3	4
3	77	16.0	7	0	88.4	24.6	31.6	1041	Oral	88.5	4	4	2
4	80	18.7	7	22	88.9	22.2	23.9	800	Tube	1060	7	7	1
5	81	22.0	7	0	87.9	25.8	28.9	1459	Oral	390	3	5	2
6	82	24.6	7	5	92.3	29.0	36.6	1188	Oral	392	4	6	2
7	83	18.5	7	9	86.6	20.3	25.2	1359	Oral	236	3	6	2
8	85	19.3	7	0	84.9	20.6	23.0	800	Tube	459	5	6	1
9	85	25.8	6	13	92.3	31.0	35.0	1345	Oral	312	2	3	3
10	86	18.8	7	3	82.5	24.0	24.8	1341	Oral	206	6	3	2
11	86	16.1	7	0	71.6	17.5	23.2	704	Oral	113	0	6	2
12	87	17.4	7	3	89.6	24.6	26.4	1342	Oral	233	1	4	2
13	89	25.5	7	21	98.3	28.6	29.8	1470	Oral	478	0	4	3
14	89	13.0	7	0	74.4	20.1	19.5	700	Tube	579	9	7	1
15	90	13.3	7	7	68.9	17.3	17.8	980	Oral	819	6	6	2
16	94	18.7	7	1	79.0	24.3	26.3	800	Tube	372	3	6	1
17	94	19.5	7	0	79.7	21.5	23.2	1397	Oral	214	5	6	2
18	95	20.9	6	14	90.2	20.6	31.0	1354	Oral	351	1	6	3
19	96	32.3	7	6	89.3	30.1	34.7	1455	Oral	227	5	4	3
20	98	19.5	6	11	98.5	23.7	30.6	1491	Oral	199	2	4	3
21	101	20.2	7	0	81.7	21.5	28.4	1180	Oral	452	3	4	2

^1^ BMI, body mass index; ^2^ MMSE, Mini Mental State Examination; ^3^ GNRI, Geriatric Nutritional Risk Index; ^4^ AC, arm circumference; ^5^ CC, calf circumference; ^6^ CAS, Constipation Assessment Scale; ^7^ BSS, Bristol Stool Scale.

## Data Availability

Sequence data are available from NCBI’s Sequence Read Archive (SRA) database (accession number PRJNA868592). All other data used for the analysis of the current study are available from the corresponding author on reasonable request.

## Supplementary Materials

The following supporting information can be downloaded at: https://www.mdpi.com/article/10.3390/nu16223839/s1, Figure S1: Correlation Heatmap of Clinical Phenotype Characteristics Among Groups; Figure S2: Correlation Matrix Heatmap of Clinical Phenotype Characteristics in Groups 1–3 (*n* = 20); Figure S3: Correlation Matrix Heatmap of Clinical Phenotype Characteristics in Groups 1–2 (*n* = 16).

## References

[1] Cabinet Office (2022). White Paper on Aging Society. https://www8.cao.go.jp/kourei/whitepaper/w-2024/html/zenbun/index.html.

[2] Statistics Bureau of Japan (2020). National Census: Population Movement Data. https://www.stat.go.jp/data/idou/index.html.

[3] Clegg A., Young J., Iliffe S., Rikkert M.O., Rockwood K. (2013). Frailty in elderly people. Lancet.

[4] Fried L.P., Tangen C.M., Walston J., Newman A.B., Hirsch C., Gottdiener J., Seeman T., Tracy R., Kop W.J., Burke G. (2001). Frailty in older adults: Evidence for a phenotype. J. Gerontol. A Biol. Sci. Med. Sci..

[5] Morley J.E., Vellas B., van Kan G.A., Anker S.D., Bauer J.M., Bernabei R., Cesari M., Chumlea W.C., Doehner W., Evans J. (2013). Frailty consensus: A call to action. J. Am. Med. Dir. Assoc..

[6] Ministry of Health, Labour and Welfare (2023). Revision of Medical Care and Long-Term Care Fees for Fiscal Year 2024. https://www.mhlw.go.jp/stf/seisakunitsuite/bunya/0000188411_00045.html.

[7] O’Toole P.W., Jeffery I.B. (2015). Gut microbiota and aging. Science.

[8] Smith M.I., Yatsunenko T., Manary M.J., Trehan I., Mkakosya R., Cheng J., Kau A.L., Rich S.S., Concannon P., Mychaleckyj J.C. (2013). Gut microbiomes of Malawian twin pairs discordant for kwashiorkor. Science.

[9] Claesson M.J., Jeffery I.B., Conde S., Power S.E., O’Connor E.M., Cusack S., Harris H.M., Coakley M., Lakshminarayanan B., O’Sullivan O. (2012). Gut microbiota composition correlates with diet and health in the elderly. Nature.

[10] Rockwood K., Theou O. (2020). Using the Clinical Frailty Scale in allocating scarce health care resources. Can. Geriatr. J..

[11] Sugishita M., Hemmi I., JADNI (2010). Validity and reliability of the Min Mental State Examination-Japanese (MMSE-J): A preliminary report. Cogn. Neurosci..

[12] Kondrup J., Allison S.P., Elia M., Vellas B., Plauth M. (2003). ESPEN guidelines for nutrition screening 2002. Clin. Nutr..

[13] Vellas B., Guigoz Y., Garry P.J., Nourhashemi F., Bennahum D., Lauque S., Albarede J.L. (1999). The Mini Nutritional Assessment (MNA) and its use in grading the nutritional state of elderly patients. Nutrition.

[14] Ministry of Health, Labour and Welfare (Japan) [Internet] (2009). Basic Questionnaire. https://www.mhlw.go.jp/file/05-Shingikai-11901000-Koyoukintoujidoukateikyoku-Soumuka/0000126242.pdf.

[15] Finkel S.I., Lyons J.S., Anderson R.L. (1992). Reliability and validity of the Cohen–Mansfield agitation inventory in institutionalized elderly. Int. J. Geriatr. Psychiatry.

[16] McMillan S.C., Williams F.A. (1989). Validity and reliability of the Constipation Assessment Scale. Cancer Nurs..

[17] Lewis S.J., Heaton K.W. (1997). Stool form scale as a useful guide to intestinal transit time. Scand. J. Gastroenterol..

[18] O’Donnell L.J., Virjee J., Heaton K.W. (1990). Detection of pseudodiarrhoea by simple clinical assessment of intestinal transit rate. BMJ.

[19] Inoue R., Ayabe M., Hiramatsu S., Sato Y., Ogawa A., Doi M., Yasmin S.A., Kageyama S., Seto C., Sumida M. (2020). Malted rice amazake ingestion changes constipation and microbiota in independently living older adults. J. Clin. Biochem. Nutr..

[20] Hosomi K., Ohno H., Murakami H., Natsume-Kitatani Y., Tanisawa K., Hirata S., Suzuki H., Nagatake T., Nishino T., Mizuguchi K. (2017). Method for preparing DNA from feces in guanidine thiocyanate solution affects 16S rRNA-based profiling of human microbiota diversity. Sci. Rep..

[21] Caporaso J.G., Kuczynski J., Stombaugh J., Bittinger K., Bushman F.D., Costello E.K., Fierer N., Peña A.G., Goodrich J.K., Gordon J.I. (2010). QIIME allows analysis of high-throughput community sequencing data. Nat. Methods.

[22] Mohsen A., Park J., Kawashima H., Chen Y., Natsume-Kitatani Y., Mizuguchi K. (2018). Auto-q Qiime Analysis Automating Script. [Internet]. Zenodo. https://github.com/Attayeb/auto-q.

[23] Quast C., Pruesse E., Yilmaz P., Gerken J., Schweer T., Yarza P., Peplies J., Glöckner F.O. (2013). The SILVA ribosomal RNA gene database project: Improved data processing and web-based tools. Nucleic Acids Res..

[24] Edgar R.C. (2010). Search and clustering orders of magnitude faster than BLAST. Bioinformatics.

[25] McMurdie P.J., Holmes S. (2013). phyloseq: An R package for reproducible interactive analysis and graphics of microbiome census data. PLoS ONE.

[26] Horio M., Imai E., Yasuda Y., Watanabe T., Matsuo S. (2013). GFR estimation using standardized serum cystatin C in Japan. Am. J. Kidney Dis..

[27] Kuzuya M. (2021). Frailty. JSPEN.

[28] Rockwood K., Song X., MacKnight C., Bergman H., Hogan D.B., McDowell I., Mitnitski A. (2005). A global clinical measure of fitness and frailty in elderly people. CMAJ.

[29] Subramanian S., Huq S., Yatsunenko T., Haque R., Mahfuz M., Alam M.A., Benezra A., DeStefano J., Meier M.F., Muegge B.D. (2014). Persistent gut microbiota immaturity in malnourished Bangladeshi children. Nature.

[30] Rockwood K., Theou O. (2015). Frailty in aging. Biological, clinical and social implications. Introduction. Interdiscip. Top. Gerontol. Geriatr..

[31] Zhang L., Liao J., Chen Q., Chen M., Kuang Y., Chen L., He W. (2020). Characterization of the gut microbiota in frail elderly patients. Aging Clin. Exp. Res..

[32] van Tongeren S.P., Slaets J.P., Harmsen H.J., Welling G.W. (2005). Fecal microbiota composition and frailty. Appl. Environ. Microbiol..

[33] Rivière A., Selak M., Lantin D., Leroy F., De Vuyst L. (2016). Bifidobacteria and butyrate-producing colon bacteria: Importance and strategies for their stimulation in the human gut. Front. Microbiol..

[34] Tedelind S., Westberg F., Kjerrulf M., Vidal A. (2007). Anti-inflammatory properties of the short-chain fatty acids acetate and propionate: A study with relevance to inflammatory bowel disease. World J. Gastroenterol..

[35] Weir T.L., Manter D.K., Sheflin A.M., Barnett B.A., Heuberger A.L., Ryan E.P. (2013). Stool microbiome and metabolome differences between colorectal cancer patients and healthy adults. PLoS ONE.

[36] Yachida S., Mizutani S., Shiroma H., Shiba S., Nakajima T., Sakamoto T., Watanabe H., Masuda K., Nishimoto Y., Kubo M. (2019). Metagenomic and metabolomic analyses reveal distinct stage-specific phenotypes of the gut microbiota in colorectal cancer. Nat. Med..

