# Supplementary material for: Clinical Phenotypes Associated with the Gut Microbiome in Older Japanese People with Care Needs in a Nursing Home
Source: Nutrients. 2024 Nov 8;16(22):3839. doi: 10.3390/nu16223839 (PMC11597083; doi:10.3390/nu16223839)
Supplement: Supplementary file 1 [file nutrients-16-03839-s001.zip › nutrients-3284257-supplementary.pptx]

## Slide 1
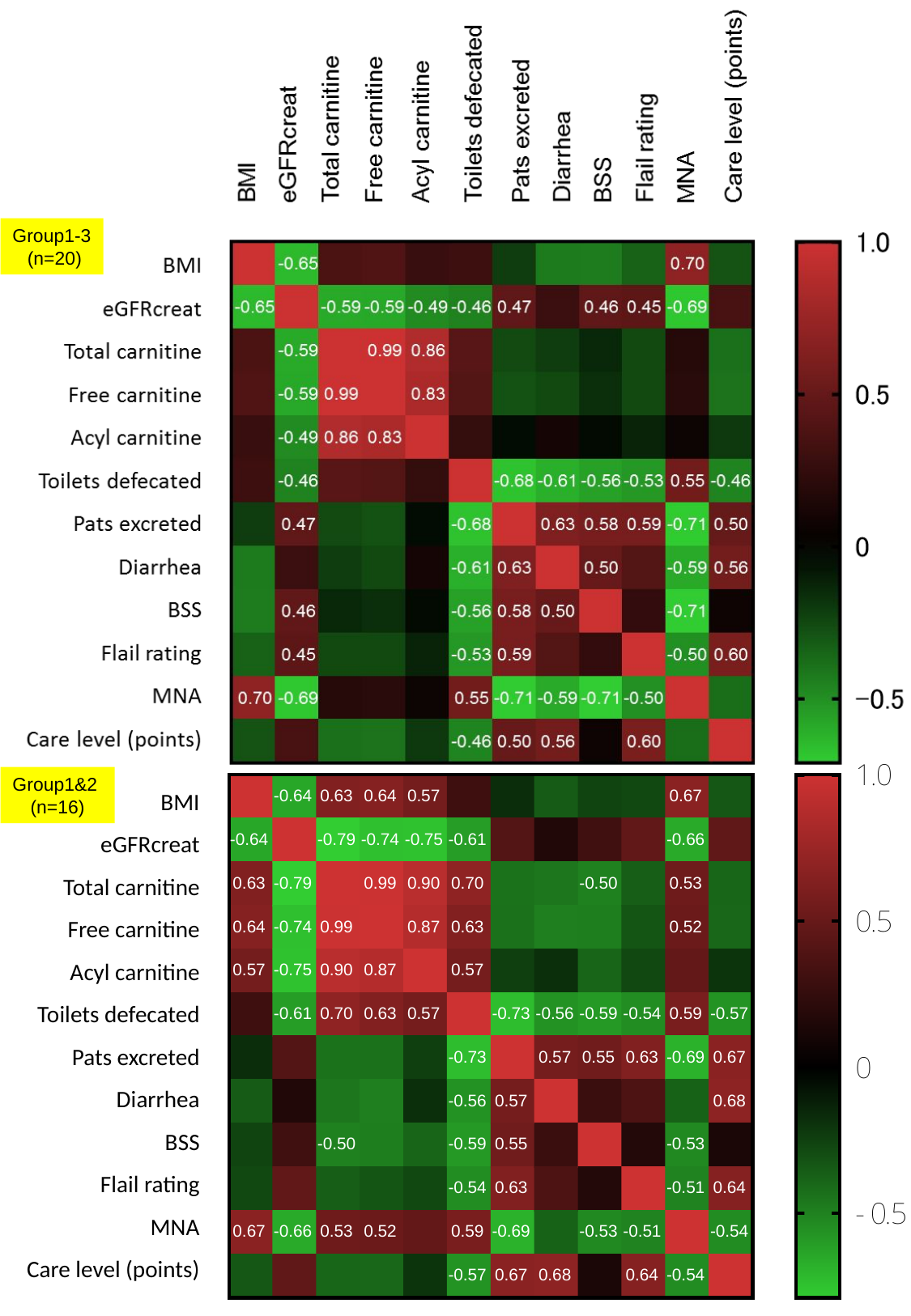

Group1-3
 (n=20)
| BMI | eGFRcreat | Total carnitine | Free carnitine | Acyl carnitine | Toilets defecated | Pats excreted | Diarrhea | BSS | Flail rating | MNA | Care level (points) |
| --- | --- | --- | --- | --- | --- | --- | --- | --- | --- | --- | --- |
Group1&2
(n=16)
BMI
eGFRcreat
Total carnitine
Free carnitine
Acyl carnitine
Toilets defecated
Pats excreted
Diarrhea
BSS
Flail rating
MNA
Care level (points)
| | -0.64 | 0.63 | 0.64 | 0.57 | | | | | | 0.67 | |
| --- | --- | --- | --- | --- | --- | --- | --- | --- | --- | --- | --- |
| -0.64 | | -0.79 | -0.74 | -0.75 | -0.61 | | | | | -0.66 | |
| 0.63 | -0.79 | | 0.99 | 0.90 | 0.70 | | | -0.50 | | 0.53 | |
| 0.64 | -0.74 | 0.99 | | 0.87 | 0.63 | | | | | 0.52 | |
| 0.57 | -0.75 | 0.90 | 0.87 | | 0.57 | | | | | | |
| | -0.61 | 0.70 | 0.63 | 0.57 | | -0.73 | -0.56 | -0.59 | -0.54 | 0.59 | -0.57 |
| | | | | | -0.73 | | 0.57 | 0.55 | 0.63 | -0.69 | 0.67 |
| | | | | | -0.56 | 0.57 | | | | | 0.68 |
| | | -0.50 | | | -0.59 | 0.55 | | | | -0.53 | |
| | | | | | -0.54 | 0.63 | | | | -0.51 | 0.64 |
| 0.67 | -0.66 | 0.53 | 0.52 | | 0.59 | -0.69 | | -0.53 | -0.51 | | -0.54 |
| | | | | | -0.57 | 0.67 | 0.68 | | 0.64 | -0.54 | |

## Slide 2
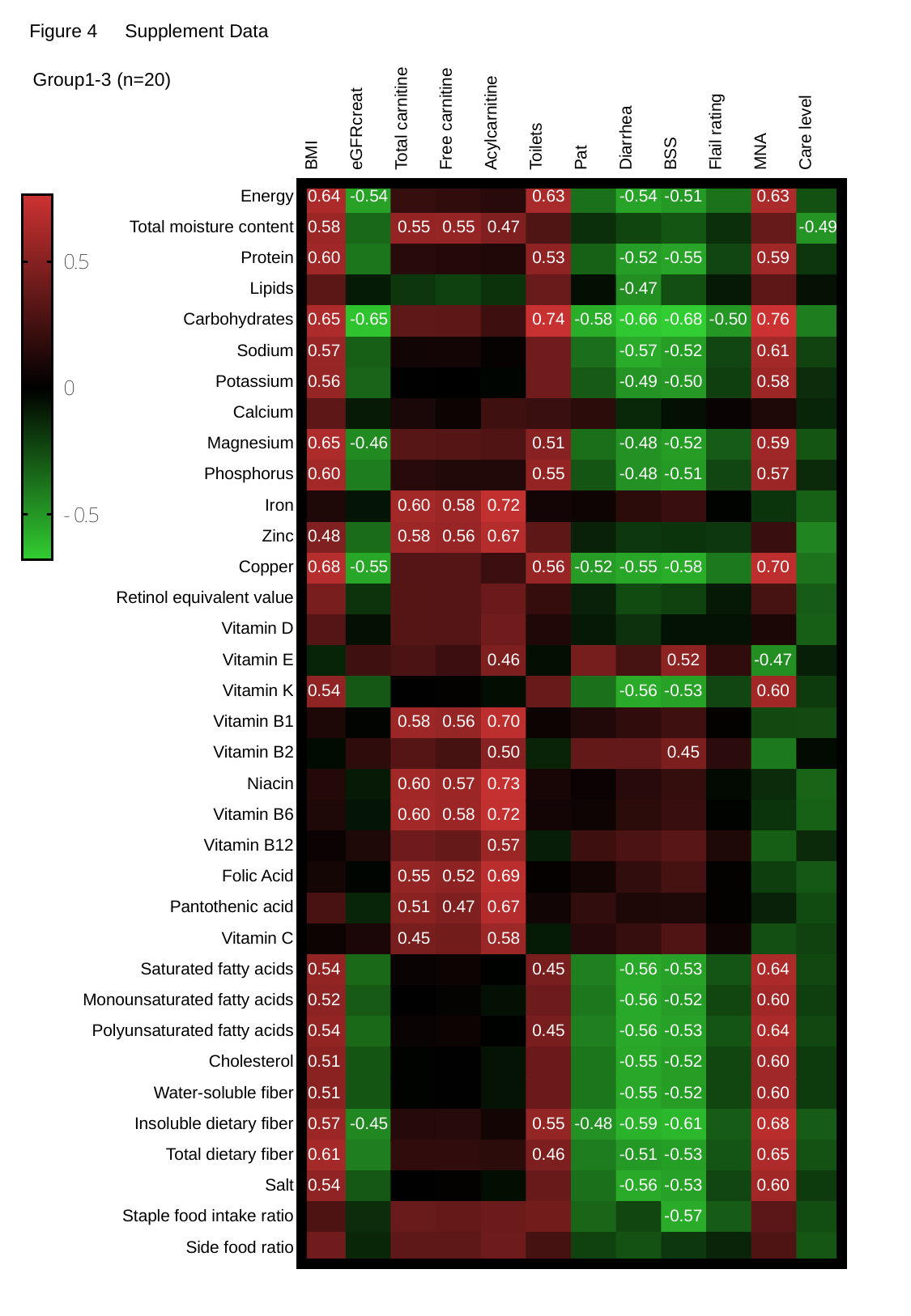

Figure 4　Supplement Data
| | BMI | eGFRcreat | Total carnitine | Free carnitine | Acylcarnitine | Toilets | Pat | Diarrhea | BSS | Flail rating | MNA | Care level |
| --- | --- | --- | --- | --- | --- | --- | --- | --- | --- | --- | --- | --- |
| Energy | 0.64 | -0.54 | | | | 0.63 | | -0.54 | -0.51 | | 0.63 | |
| Total moisture content | 0.58 | | 0.55 | 0.55 | 0.47 | | | | | | | -0.49 |
| Protein | 0.60 | | | | | 0.53 | | -0.52 | -0.55 | | 0.59 | |
| Lipids | | | | | | | | -0.47 | | | | |
| Carbohydrates | 0.65 | -0.65 | | | | 0.74 | -0.58 | -0.66 | -0.68 | -0.50 | 0.76 | |
| Sodium | 0.57 | | | | | | | -0.57 | -0.52 | | 0.61 | |
| Potassium | 0.56 | | | | | | | -0.49 | -0.50 | | 0.58 | |
| Calcium | | | | | | | | | | | | |
| Magnesium | 0.65 | -0.46 | | | | 0.51 | | -0.48 | -0.52 | | 0.59 | |
| Phosphorus | 0.60 | | | | | 0.55 | | -0.48 | -0.51 | | 0.57 | |
| Iron | | | 0.60 | 0.58 | 0.72 | | | | | | | |
| Zinc | 0.48 | | 0.58 | 0.56 | 0.67 | | | | | | | |
| Copper | 0.68 | -0.55 | | | | 0.56 | -0.52 | -0.55 | -0.58 | | 0.70 | |
| Retinol equivalent value | | | | | | | | | | | | |
| Vitamin D | | | | | | | | | | | | |
| Vitamin E | | | | | 0.46 | | | | 0.52 | | -0.47 | |
| Vitamin K | 0.54 | | | | | | | -0.56 | -0.53 | | 0.60 | |
| Vitamin B1 | | | 0.58 | 0.56 | 0.70 | | | | | | | |
| Vitamin B2 | | | | | 0.50 | | | | 0.45 | | | |
| Niacin | | | 0.60 | 0.57 | 0.73 | | | | | | | |
| Vitamin B6 | | | 0.60 | 0.58 | 0.72 | | | | | | | |
| Vitamin B12 | | | | | 0.57 | | | | | | | |
| Folic Acid | | | 0.55 | 0.52 | 0.69 | | | | | | | |
| Pantothenic acid | | | 0.51 | 0.47 | 0.67 | | | | | | | |
| Vitamin C | | | 0.45 | | 0.58 | | | | | | | |
| Saturated fatty acids | 0.54 | | | | | 0.45 | | -0.56 | -0.53 | | 0.64 | |
| Monounsaturated fatty acids | 0.52 | | | | | | | -0.56 | -0.52 | | 0.60 | |
| Polyunsaturated fatty acids | 0.54 | | | | | 0.45 | | -0.56 | -0.53 | | 0.64 | |
| Cholesterol | 0.51 | | | | | | | -0.55 | -0.52 | | 0.60 | |
| Water-soluble fiber | 0.51 | | | | | | | -0.55 | -0.52 | | 0.60 | |
| Insoluble dietary fiber | 0.57 | -0.45 | | | | 0.55 | -0.48 | -0.59 | -0.61 | | 0.68 | |
| Total dietary fiber | 0.61 | | | | | 0.46 | | -0.51 | -0.53 | | 0.65 | |
| Salt | 0.54 | | | | | | | -0.56 | -0.53 | | 0.60 | |
| Staple food intake ratio | | | | | | | | | -0.57 | | | |
| Side food ratio | | | | | | | | | | | | |
Group1-3 (n=20)

## Slide 3
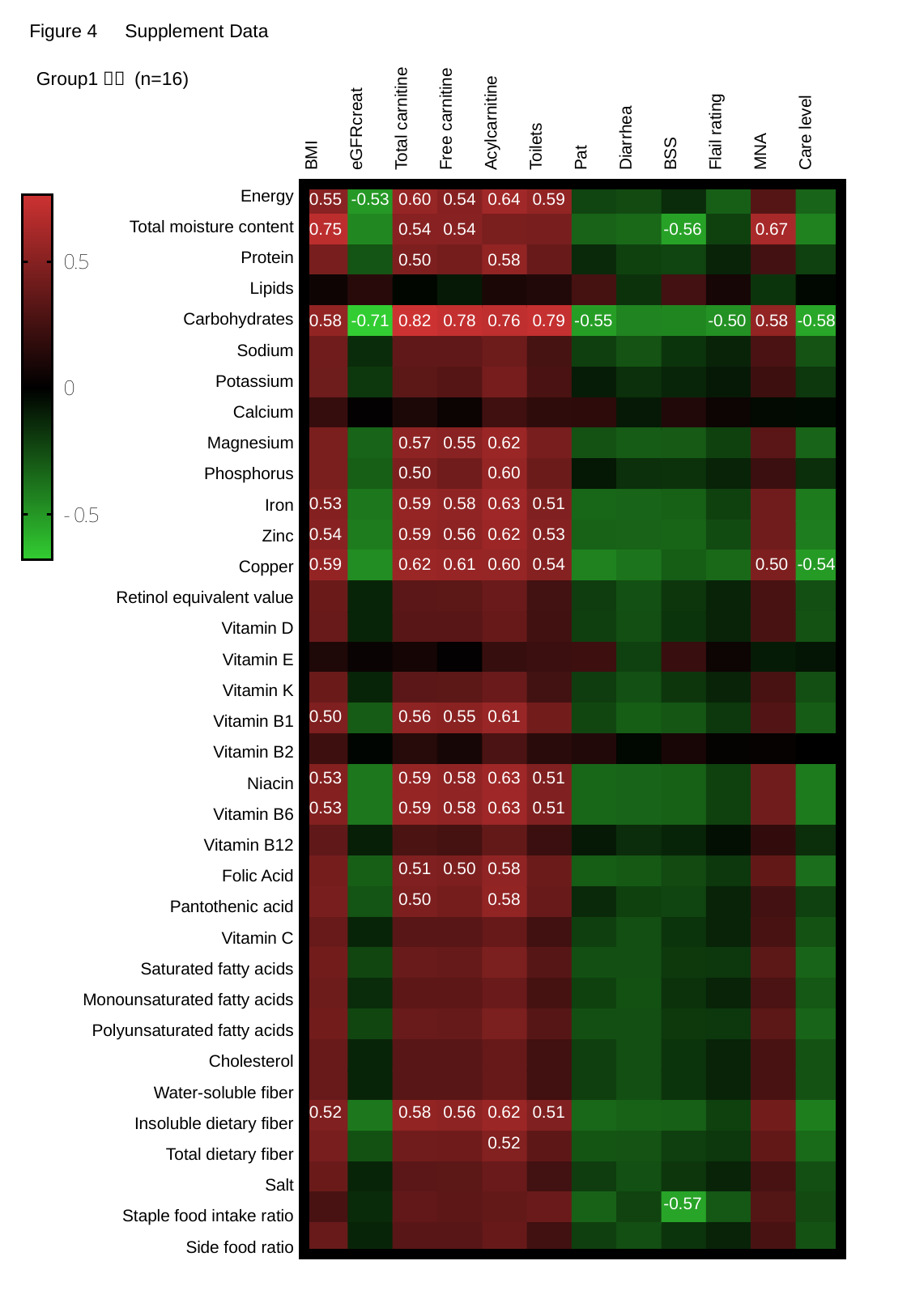

Figure 4　Supplement Data
Group1＆２ (n=16)
| | BMI | eGFRcreat | Total carnitine | Free carnitine | Acylcarnitine | Toilets | Pat | Diarrhea | BSS | Flail rating | MNA | Care level |
| --- | --- | --- | --- | --- | --- | --- | --- | --- | --- | --- | --- | --- |
| Energy | | | | | | | | | | | | |
| Total moisture content | | | | | | | | | | | | |
| Protein | | | | | | | | | | | | |
| Lipids | | | | | | | | | | | | |
| Carbohydrates | | | | | | | | | | | | |
| Sodium | | | | | | | | | | | | |
| Potassium | | | | | | | | | | | | |
| Calcium | | | | | | | | | | | | |
| Magnesium | | | | | | | | | | | | |
| Phosphorus | | | | | | | | | | | | |
| Iron | | | | | | | | | | | | |
| Zinc | | | | | | | | | | | | |
| Copper | | | | | | | | | | | | |
| Retinol equivalent value | | | | | | | | | | | | |
| Vitamin D | | | | | | | | | | | | |
| Vitamin E | | | | | | | | | | | | |
| Vitamin K | | | | | | | | | | | | |
| Vitamin B1 | | | | | | | | | | | | |
| Vitamin B2 | | | | | | | | | | | | |
| Niacin | | | | | | | | | | | | |
| Vitamin B6 | | | | | | | | | | | | |
| Vitamin B12 | | | | | | | | | | | | |
| Folic Acid | | | | | | | | | | | | |
| Pantothenic acid | | | | | | | | | | | | |
| Vitamin C | | | | | | | | | | | | |
| Saturated fatty acids | | | | | | | | | | | | |
| Monounsaturated fatty acids | | | | | | | | | | | | |
| Polyunsaturated fatty acids | | | | | | | | | | | | |
| Cholesterol | | | | | | | | | | | | |
| Water-soluble fiber | | | | | | | | | | | | |
| Insoluble dietary fiber | | | | | | | | | | | | |
| Total dietary fiber | | | | | | | | | | | | |
| Salt | | | | | | | | | | | | |
| Staple food intake ratio | | | | | | | | | | | | |
| Side food ratio | | | | | | | | | | | | |
| 0.55 | -0.53 | 0.60 | 0.54 | 0.64 | 0.59 | | | | | | |
| --- | --- | --- | --- | --- | --- | --- | --- | --- | --- | --- | --- |
| 0.75 | | 0.54 | 0.54 | | | | | -0.56 | | 0.67 | |
| | | 0.50 | | 0.58 | | | | | | | |
| | | | | | | | | | | | |
| 0.58 | -0.71 | 0.82 | 0.78 | 0.76 | 0.79 | -0.55 | | | -0.50 | 0.58 | -0.58 |
| | | | | | | | | | | | |
| | | | | | | | | | | | |
| | | | | | | | | | | | |
| | | 0.57 | 0.55 | 0.62 | | | | | | | |
| | | 0.50 | | 0.60 | | | | | | | |
| 0.53 | | 0.59 | 0.58 | 0.63 | 0.51 | | | | | | |
| 0.54 | | 0.59 | 0.56 | 0.62 | 0.53 | | | | | | |
| 0.59 | | 0.62 | 0.61 | 0.60 | 0.54 | | | | | 0.50 | -0.54 |
| | | | | | | | | | | | |
| | | | | | | | | | | | |
| | | | | | | | | | | | |
| | | | | | | | | | | | |
| 0.50 | | 0.56 | 0.55 | 0.61 | | | | | | | |
| | | | | | | | | | | | |
| 0.53 | | 0.59 | 0.58 | 0.63 | 0.51 | | | | | | |
| 0.53 | | 0.59 | 0.58 | 0.63 | 0.51 | | | | | | |
| | | | | | | | | | | | |
| | | 0.51 | 0.50 | 0.58 | | | | | | | |
| | | 0.50 | | 0.58 | | | | | | | |
| | | | | | | | | | | | |
| | | | | | | | | | | | |
| | | | | | | | | | | | |
| | | | | | | | | | | | |
| | | | | | | | | | | | |
| | | | | | | | | | | | |
| 0.52 | | 0.58 | 0.56 | 0.62 | 0.51 | | | | | | |
| | | | | 0.52 | | | | | | | |
| | | | | | | | | | | | |
| | | | | | | | | -0.57 | | | |
| | | | | | | | | | | | |
